# Role of Platelet Cytokines in Dengue Virus Infection

**DOI:** 10.3389/fcimb.2020.561366

**Published:** 2020-09-30

**Authors:** Anamika Singh, Piyush Bisht, Sulagna Bhattacharya, Prasenjit Guchhait

**Affiliations:** ^1^Disease Biology Laboratory, Regional Center for Biotechnology, National Capital Region Biotech Science Cluster, Faridabad, India; ^2^School of Biotechnology, Kalinga Institute of Industrial Technology, Bhubaneswar, India

**Keywords:** platelets, cytokines, PF4, immune modulation, dengue infection

## Abstract

Platelets are anucleated blood cells derived from bone marrow megakaryocytes and play a crucial role in hemostasis and thrombosis. Platelets contain specialized storage organelles, called alpha-granules, contents of which are rich in cytokines such as C-X-C Motif Chemokine Ligand (CXCL) 1/4/7, (C-C motif) ligand (CCL) 5/3, CXCL8 (also called as interleukin 8, IL-8), and transforming growth factor β (TGF-β). Activation of platelets lead to degranulation and release of contents into the plasma. Platelet activation is a common event in many viral infections including human immunodeficiency virus (HIV), H1N1 influenza, Hepatitis C virus (HCV), Ebola virus (EBV), and Dengue virus (DENV). The cytokines CXCL8, CCL5 (also known as Regulated on Activation, Normal T Expressed and Secreted, RANTES), tumor necrosis factor α (TNF-α), CXCL1/5 and CCL3 released, promote development of a pro-inflammatory state along with the recruitment of other immune cells to the site of infection. Platelets also interact with Monocytes and Neutrophils and facilitate their activation to release different cytokines which further enhances inflammation. Upon activation, platelets also secrete factors such as CXCL4 (also known as platelet factor, PF4), CCL5 and fibrinopeptides which are critical regulators of replication and propagation of several viruses in the host. Studies suggest that CXCL4 can both inhibit as well as enhance HIV1 infection. Data from our lab show that CXCL4 inhibits interferon (IFN) pathway and promotes DENV replication in monocytes *in vitro* and in patients significantly. Inhibition of CXCL4 mediated signaling results in increased IFN production and suppressed DENV and JEV replication in monocytes. In this review, we discuss the role of platelets in viral disease progression with a focus on dengue infection.

## Dengue Infection

Dengue is one of the most prevalent arboviral diseases (Simmons et al., 2012) affecting ~390 million people around the globe (Bhatt et al., 2013) causing an estimated 20000 deaths each year (Simon et al., 2015). Mainly this virus affects the tropical and subtropical regions of the world (Wilder-Smith et al., 2019). India accounts for the largest number of dengue cases, with ~33 million apparent and another 100 million asymptomatic infections occurring annually (Bhatt et al., 2013). Since the 1990s, dengue epidemics have become recurrent in several parts of India, at a rate of 34.81 per million of the population in 2010–2014. Thus, indicating that the number of dengue cases has increased markedly in recent times (Mutheneni et al., 2017). The principle vectors for transmission of the disease are mosquitoes *Aedes aegypti* and *Aedes albopictus* (Kraemer et al., 2015). Whereas, non-vector transmission of the virus is also possible through blood transfusion, organ transplantation, needle stick injuries and mucosal splashes (Wilder-Smith et al., 2009; Busch et al., 2016; Sabino et al., 2016). Dengue virus (DENV) is a Flavivirus that belongs to the family Flaviviridae and has four serotypes, DENV1–4. The virus genome consists of a single-strand of positive-polarity RNA which codes for three structural proteins (capsid C, membrane M, and the envelope E) and seven non-structural proteins (NS1, NS2A, NS2B, NS3, NS4A, NS4B, NS5) (Noble and Shi, 2012). The clinical features of the disease range from asymptomatic infection, undifferentiated febrile illness (dengue fever) to severe complication including Dengue Haemorrhagic Fever (DHF) and Dengue Shock Syndrome (DSS), characterized by plasma leakage and coagulopathy (Kalayanarooj, 2011; Simmons et al., 2012). The pathogenesis of severe dengue is the result of a complex interaction between viral and host factors. The four DENV serotypes (DENV 1–4) share homology, but each serotype possesses specific immunoreactivity. Primary infection with one DENV serotype increases the risk of severe dengue upon secondary infection with other DENV serotype. This is because the sub-neutralizing cross-reactive antibodies opsonize mature and immature virus particles, causing infection of mononuclear phagocytes via Fc-receptor (FcR) thus enhancing DENV infection, termed as antibody dependent enhancement (ADE) (Littaua et al., 1990; Dejnirattisai et al., 2010; Schmidt, 2010; Guzman et al., 2013). This is characterized by persistent high fever, thrombocytopenia, event of DHF or DSS and elevated IgG/IgM ratio (Innis et al., 1989; Shu et al., 2003; Prince et al., 2011; Cucunawangsih and Kurniawan, 2015). Complement activation by virus-antibody complexes and T-cell mediated immune response have also been reported in the progression of secondary dengue infection. Independent of primary or secondary infection, platelet activation is the hallmark of dengue infection. Thrombocytopenia (decrease in platelet count) is another common symptom of dengue infection and is associated with hemodynamic instability and progression in severity of dengue fever (Krishnamurti et al., 2001; Schexneider and Reedy, 2005; Mourão et al., 2007; Bozza et al., 2008).

## Platelet Functions

The primary and most important role of platelets is to maintain hemostasis (Clemetson, 2012). Platelets are mediators of thrombosis too (Kaplan and Jackson, 2011). Besides, platelets also contribute to non-canonical functions such as immune modulation, atherogenesis, tissue repair and regeneration, angiogenesis, and metastasis (Lindemann et al., 2007; Nurden, 2011; Semple et al., 2011; Eisinger and Langer, 2018; Koupenova et al., 2018; Schlesinger, 2018). Platelets mediate innate immune response and contribute to antimicrobial activity either by releasing antimicrobial proteins or by modulating immune response of other immune cells such as neutrophils and monocytes (Flad and Brandt, 2010). Platelet dysfunction can lead to impaired immune or inflammatory responses and tissue damage. During infection, platelet activation can lead to serious pathophysiological conditions such as Infective Endocarditis and Disseminated Intravascular Coagulation (Beynon et al., 2006; Kitchens, 2009). Platelet activation followed by thrombocytopenia is found to be associated with patients of several infections and sepsis (Akca et al., 2002; Claushuis et al., 2016; Levi, 2016). Platelet activation is the hallmark of many viral infections including dengue, HIV, HCV, H1N1, and Ebola (Geisbert et al., 2003; Chaipan et al., 2006; Afdhal et al., 2008; Assinger et al., 2014; Ojha et al., 2017, 2019).

## Platelet Granules and Cytokines

Platelets contain α and dense granules, also known as storage granules. These granules store huge numbers of proteins, RNAs, antimicrobial peptides and growth factors (Manne et al., 2017). α granules are ~500 nm in diameter, round in shape and around 50 granules are present in each platelet (Yadav and Storrie, 2017). Different cytokines, growth factors and antimicrobial peptides present are listed in Table 1. Upon platelet activation the α-granules release these contents into the plasma (Jonnalagadda et al., 2012). The dense granules on the other hand are smaller in size and contain Adenosine nucleotides (ATP, ADP), serotonin, calcium, magnesium and pyrophosphate (Rendu and Brohard-Bohn, 2001).

**Table 1:** Contents of platelet granules.

**Granules**	**Contents**	**Function**
α-granules Chemokines	CXCL1 (GRO-α), CXCL4 (PF4), CXCL4L1 (PF4alt), CXCL5 (ENA-78), CXCL7 (PBP), CXCL8 (IL-8), CXCL12 (SDF-1α), CXCL14 (BRAK) CCL2 (MCP-1), CCL3 (MIP-1α), CCL5 (RANTES), CCL7 (MCP-3), CCL8 (MCP-2), CCL15, CCL17 (TARC), CCL18 (PARK)	Chemotactic for different immune cells, modulate activation and differentiation of Monocytes, regulate thrombopoiesis, hematopoiesis, and T cell development
Growth factors	Epidermal Growth Factor (EGF), Hepatocyte Growth Factor (HGF), Transforming Growth Factor-β (TGF-β), Insulin like Growth Factor (IGF), Platelet derived Growth Factor (PDGF), Vascular endothelium growth Factor (VEGF)	Regulates growth
Microbicidal proteins	Thymosin-β4, Thrombocidins 1 and 2, Kinocidins, Defensins 1 and 2	Antimicrobial peptides
Dense granules	Bioactive amines (serotonin, histamine), Phosphates (polyphosphate, pyrophosphate), cations, ADP, ATP.	Hemostasis, thrombosis
Lysosome	Protein degrading enzyme (collagenase, elastase, cathepsins), carbohydrate degrading enzyme (glucosidase, galactosidase), phosphatases.	Degradation

*Blair and Flaumenhaft (2009); Karshovska et al. (2013); Heijnen and van der Sluijs (2015); Gremmel et al. (2016)*.

## Immune Function of Platelets

Platelets are modulators of innate immunity and possess both surface and intracellular receptors for detection of pathogens in the bloodstream. Receptors include Toll-like receptors (TLRs), NOD-like receptors (NLR), C-type Lectins and integrins for detection of pathogen associated molecular patterns (PAMPs), and Fc receptors and complement receptors for antibody opsonized pathogens (McDonald and Dunbar, 2019). Platelets express TLR 1–4, 6, 7, and 9 (Hamzeh-Cognasse et al., 2018; McDonald and Dunbar, 2019). On activation the TLRs promote secretion of interferons and proinflammatory cytokines such as IL-6, CXCL8, TNF-α, and CCL5 which further activate other cells resulting in increased inflammation. CD40L, expressed on platelets as a result of activation (Henn et al., 1998), mediates its interaction with CD40 present on B cells, Monocytes, Dendritic cells, Macrophages, endothelial cells and modulate their activity. CD40 and CD40L interaction leads to recruitment of TNF receptor associated factors (TRAFs) which further lead to activation of canonical and non-canonical NF-kB pathways (Bishop et al., 2007). Platelets via CD40L induce B cell isotype switching and augment CD8+ T cell response (Elzey et al., 2003). Activated platelets cause Dendritic cell maturation and activation by direct surface contact through CD40L or through soluble effectors (Czapiga et al., 2004; Hagihara et al., 2004; Martinson et al., 2004). In Dendritic cells CD40 expression tends to increase the expression of MHC class II along with essential co-stimulatory molecules such as CD58, CD80, and CD86. This further leads to improved T cell activation by better presentation of the antigen. Platelets possess an active proteasome and can process and present exogenous antigens which has been shown both *in vitro* and *in vivo* using Experimental Cerebral Malaria (ECM) mouse model (Chapman et al., 2012). An increase in surface expression of HLA class 1 has been observed on platelets obtained from patients with Dengue infection (Trugilho et al., 2017). However, the actual mode of presentation of DENV antigen still needs to be investigated.

Recently it has been shown that platelets can differentiate between bacterial LPS isoforms and as a result between pathogens (Berthet et al., 2012). This helps in providing a pathogen specific response by secreting different cytokines. It has been shown that platelets upon activation with thrombin trigger complement activation. Complement factor C3b bound to bacteria gets detected by GPIb receptor on platelet surface resulting in phagocytosis of the bacteria-platelet complex (Verschoor et al., 2011). Platelets also respond to Damage Associated Molecular Patterns (DAMPs) (Fuchs et al., 2011).

Platelets get activated upon interacting with pathogens directly through their surface receptors or indirectly through plasma proteins such as fibrinogen, vWF, complement and IgG (Fitzgerald et al., 2006; Cox et al., 2011). Upon activation by bacterial pathogens platelets release antimicrobial substances such as ROS (reactive oxygen species), antimicrobial peptides, defensins, thrombocidins and proteases (Suzuki et al., 2001; Tang et al., 2002; Trier et al., 2008; Wiesner and Vilcinskas, 2010; Yeaman, 2014). Platelets support phagocytosis and intracellular killing of bacteria by Neutrophils and release of Neutrophil Extracellular Traps (NETs) (Miyabe et al., 2004; Assinger et al., 2011; Kim and Jenne, 2016). In disease conditions such as malaria, platelets have a direct lethal effect on all major *Plasmodium species* (Kho et al., 2018). Platelets bind with erythrocytes and cause killing of intraerythrocytic *Plasmodium* along with intracellular accumulation of platelet CXCL4 (McMorran et al., 2012). In patients with *mycobacterium tuberculosis*, infected platelets along with other immune cells are responsible for pulmonary inflammation and tissue damage leading to morbidity and spread of infection (Fox et al., 2018). Thus, platelets help to fight infection but at times can also help in disease progression.

## Immune Response of Platelets During Viral Infections

CXCL4 is highly abundant in platelets. CXCL4 can associate with CCL5 to modulate monocyte functions. CCL3, CCL5, CCL7, CCL17, CXCL1, CXCL5, and CXCL8 are some of the chemokines among the whole pool of chemokines released by platelets which attract leukocytes causing further activation of platelets (Gear and Camerini, 2003). A comparative study reports that CXCL4 in conjunction with M-CSF supports HIV-1 replication in immune cells including monocytes and macrophages. It was found that CXCL4 derived macrophages can be infected with macrophage-tropic HIV-1 that uses either CCR5 or CXCR4 as a co-receptor for viral entry. CXCL4 increases HIV-1 replication in M-CSF-derived human macrophages after virus adsorption on to the cells (Schwartzkopff et al., 2009). CXCL4 has also been identified as a broad spectrum inhibitor of HIV-1 (Auerbach et al., 2012). In another study, it was found that CXCL4 inhibitory effect lies in a concentration range where it exists in monomeric form. As a monomer it binds with the viral envelope protein resulting in inhibition of HIV-1 attachment to the cell surface. But oligomerization initiated upon increasing concentration; the tetrameric or higher-order forms promoted viral replication *in vitro* (Parker et al., 2016). CXCL4 and beta thromboglobulin (β-TG) have been reported as prognostic markers in Severe Acute Respiratory Syndrome Coronavirus (SARS-CoV) infections. A decreased serum CXCL4 and increased β-TG were reported in the study (Poon et al., 2012). CXCL4 is very well-known for its regulatory functions in immune response and inflammation. Role of CXCL4 has been studied in pulmonary influenza infection, and it was found that it helps in protecting the mice from H1N1 virus infection (Guo et al., 2017). CXCL4 up-regulated in HCV induces liver fibrosis both *in vitro* and *in vivo* (Zaldivar et al., 2010). CXCL4 also increases replication and propagation of dengue and Japanese encephalitis viruses (JEV) in monocytes by downregulating the type-I interferon production, thus leading to increased viral load (Ojha et al., 2019).

Platelet derived CCL5 and CCL3 have been reported as major HIV-suppressive factors (Cocchi et al., 1995). CCL5 is also reported as inhibitor of Influenza A (Wareing et al., 2004) and HCV infection (Katsounas et al., 2011). CCL5 is reported to be involved in viral lung diseases (Culley et al., 2006). CCL17 is released by activated platelets and functions in further platelet activation in an autocrine manner by binding to its receptor CCR4 present on platelets (Gear and Camerini, 2003). A recent study reported that co-culture of monocyte derived dendritic cells (MoDCs) and HCV infected cells lead to the expression of CCL17 and CCL22 which attract regulatory T cells at the site of infection (Riezu-Boj et al., 2011). Co-culture of MoDCs with HBV transfected cells induces the expression of CCL17 and CCL22 which recruits IL-17 secreting T-cells (Zhang et al., 2020).

## Immune Response of Platelets to Dengue Infection

### Dengue Infection in Platelets

The presence of DENV has been reported in circulating platelets of dengue patients (Noisakran et al., 2009). Platelet activation and thrombocytopenia are the hallmarks of dengue infection. Platelet activation results in the release of the granular constituents. Platelets from dengue patients present signs of activation, mitochondrial dysfunction and activation of apoptosis caspase cascade resulting in thrombocytopenia. Dendritic cell-specific intercellular adhesion molecule-3-grabbing non-integrin (DC-SIGN) also termed as CD209 has been shown as a critical receptor involved in DENV-dependent platelet activation (Hottz et al., 2013b). DENV directly binds to the surface receptors DC-SIGN, heparan sulfate proteoglycan (HSP) and CLEC-2 (C-type-lectin-like receptor 2) on platelets and replicates inside the platelets (Simon et al., 2015; Sung and Hsieh, 2019). One of the findings suggests that platelets attach to the histones (H2A) present in systemic circulation of the dengue patients, which lead to their activation (Trugilho et al., 2017). Platelets have specialized FcγRII receptors for binding of immunoglobulin coated virus particles. Antibody dependent enhancement of DENV occurs via these FcγRII receptors on platelets (Wang et al., 1995) upon secondary dengue infection. This leads to platelet activation and thrombocytopenia which results into severe dengue diseases, DHF, and DSS.

### Immunoregulation of Dengue Infection by Platelets

Platelets are the key players involved in immunoregulation of dengue disease as there is platelet activation upon dengue infection and release of the granular contents. Cytokines are released from alpha granules, and have been shown to play regulatory roles in dengue virus infection. The CCR1-CCL2 axis plays an important role in the pathogenesis of dengue infection whereas CCR1-CCL5 axis was found to show a protective role in dengue infection (Sierra et al., 2014). A study has suggested that low levels of CCL5 and high levels of CXCL8 during early dengue infection could serve as a marker for severe dengue disease (Patra et al., 2019). Upon DENV infection there is activation of endothelial cells leading to increased expression of E-selectin on the endothelial cells. E-selectin and P-selectin helps in platelet adhesion to endothelial cells (Krishnamurti et al., 2002). P-selectin expressed on the surface of activated platelets promote interaction of platelets with monocytes and neutrophils leading to aggregate formation (Onlamoon et al., 2010). The endothelial cells upon dengue infection secrete CXCL8, IL-6, CXCL10, CXCL11, and CCL5 (Avirutnan et al., 1998; Dalrymple and Mackow, 2012; Kelley et al., 2012). Platelets also secrete IL-1β, CXCL8, and CCL5 and contribute to the total cytokine pool. These cytokines together help in increasing the vascular permeability and possess chemoattractant properties which progress inflammation and plasma leakage *in vivo* thus leading to dengue disease severity (Kelley et al., 2012).

Increased platelet monocyte aggregates have been reported in dengue patients having thrombocytopenia and increased vascular permeability. Platelet binding modulates cytokine release by monocytes in dengue infection. Interaction of platelets isolated from dengue patients with monocytes from healthy individuals lead to the synthesis and secretion of IL-1β, CXCL8, and IL-10 by the monocytes. Also interaction of monocytes with apoptotic platelets leads to secretion of IL-10 through P selectin and phosphatidylserine recognition in platelet-monocyte aggregates. IL-10 secretion requires platelet-monocyte contact but not phagocytosis of platelets by the monocytes. Activated and apoptotic platelets aggregate with monocytes during dengue infection and cause specific cytokine responses contributing to the pathogenesis of dengue. The cytokines IL-1β, CXCL8, and IL-10, released by monocytes in response to interactions with platelets from dengue patients, are frequently observed to be increased in plasma of severe dengue patients (Hottz et al., 2014).

Vascular leakage is one of the hallmarks of DHF, and an increased CCL2 level is reported in DHF. CCL2 leads to disrupted distribution of tight junction protein on the cell membrane of human umbilical vein endothelial cells (HUVEC) leading to increased vascular permeability (Lee et al., 2006). Platelets release IL-1β upon DENV infection by recruiting nucleotide-binding domain leucine rich repeat containing protein (NLRP3) inflammasome and caspase-1 which is highly correlated with increased vascular permeability and activation of innate immunity in dengue infection (Hottz et al., 2013a, 2014; Guabiraba and Ryffel, 2014).

Macrophage migration inhibitory factor (MIF) is a pleiotropic proinflammatory cytokine that mediates several immune responses, serum levels of MIF, IL-6, and IL-10 are reported to be higher in DHF patients as compared to DF patients (Chen et al., 2006). MIF is secreted mainly by T cells, and also by other immune and non-immune cells such as macrophages, endothelial cells, epithelial cells, neutrophils and monocytes (Lai et al., 2020). It is also secreted by platelets (Wirtz et al., 2015). Mif–/– mice show lesser DENV induced inflammation, thrombocytopenia and viral load suggesting it as one of the crucial cytokines involved in dengue pathogenesis (Assunção-Miranda et al., 2010). MIF helps in enhancing dengue viral replication in host cells, it also causes endothelial hyperpermeability upon DENV infection, and also modulates the functions of immune cells. MIF inhibition during dengue infection also leads to decreased TNF-α and IL-6 production which otherwise lead to vascular hyperpermeability. MIF release by neutrophils leads to neutrophil extracellular trap (NET) formation and inflammation which further helps in dengue pathogenesis. It can also be a potential therapeutic target against dengue infection (Lai et al., 2020). In comparison with DENV infection, secretory NS1 protein released upon DENV infection and exogenous NS1 protein stimulation also leads to a partial inflammatory phenotype in platelets. Similar to infected platelets, NS1-stimulated platelets also release the stored cytokines/chemokines CXCL4, CCL5, and MIF but, in contrast, they do not secrete IL-1β. NS1 induces pro-IL-1β synthesis in platelets but does not induce caspase-1 activation for IL-1β processing and secretion, which occurs upon classical DENV infection providing all necessary signals for IL-1β synthesis, caspase-1 activation, and IL-1β release. There is increased caspase-1 activation in platelets stimulated with either ATP, NS1, and ATP, or DENV, but not in platelets stimulated with NS1 alone (Quirino-Teixeira et al., 2020). DENV activated platelets deliver inflammatory signals to monocytes leading to secretion of CXCL8, IL-10, and IL-6, and also show increased accumulation of lipid droplets (LD) in the monocytes after 18 h of interaction. Stimuli-specific-activated platelets can cause phenotypic changes and metabolic reprogramming in monocytes. Activated platelets exposed to DENV *in vitro* form aggregates with monocytes and signal to LD formation and CXCL8, IL-10, CCL2 and prostaglandin E2 (PGE2) secretion. Pharmacologic inhibition of LD biogenesis prevents PGE2 secretion, but not CXCL8 release, by platelet-monocyte complexes. Mechanistically LD formation in monocytes exposed to DENV-activated platelets is partially dependent on platelet-produced MIF. LD formation is higher in monocytes, which have platelets adhered on their surface, suggesting the importance of adhesion besides paracrine signaling. Activated platelets aggregate with monocytes during DENV infection and lead to LD biogenesis and release of inflammatory mediators (Barbosa-Lima et al., 2020).

### CXCL4 in Dengue Infection

CXCL4 is the most abundant chemokine present in platelet α granules, released upon platelet activation (Gleissner et al., 2008). A quantitative proteomic study has investigated the protein content of platelets in dengue patient samples and healthy controls. In dengue patients, activated platelets release a significant amount of CXCL4 in plasma as compared to healthy volunteers (Trugilho et al., 2017). CXCL4 has been identified as a prognostic tool for classifying acute and severe dengue patients (Fragnoud et al., 2015). A comparative study between dengue and leptospirosis (caused by *Leptospira* bacteria) revealed that out of the 19 biomarkers assessed CXCL4 was the one higher in dengue fever compared to leptospirosis (Conroy et al., 2014). A recent work from our lab also showed that CXCL4 is one of the abundant proteins present in blood plasma upon dengue infection. It also helps in propagating the virus inside monocytes *in vitro*. Binding of CXCL4 to CXCR3 leads to increased phosphorylation of p38-MAPK and diminished activation of STAT-2 and IRF-9. This decreases the synthesis and secretion of IFN-α by the DENV2 infected monocytes, resulting in 3–4-fold increase in virus replication. Also, blocking CXCL4 using neutralizing CXCL4 antibodies or its receptor CXCR3 through inhibitor AMG487 reversed the above signaling pathway and significantly restored the IFN-α production, thus inhibiting the DENV propagation in monocytes. The study also showed a decrease in the levels of proinflammatory cytokines TNF-α, IL-1β, and IL-6 in monocytes upon treatment of the cells with rhCXCL4, which was rescued upon treating the cells with anti-CXCL4 antibody or antagonist AMG487, concluding that CXCL4 significantly impacts dengue virus replication (Ojha et al., 2019).

### Therapy for Dengue

Till date, there is no specific treatment for Dengue infection. Although few studies describe the development of drugs targeting host factors required for DENV propagation. Chloroquine (CQ) has immunomodulatory effects by suppressing release of TNF-α and IL-6 (Tricou et al., 2010). Although the *in vitro* study showed some promising results but a randomized trial in patients did not show significant reduction in the development of DHF (Savarino et al., 2003). Another drug, Celgosivir is an alkaloid castanospermine derived from the Moreton Bay Chestnut tree showed some inhibitory effects on all four serotypes (Durantel, 2009). Celgosivir was tested in Phase I and II trials as a possible treatment for HIV and hepatitis C infection and was found to be safe (Durantel, 2009; Rathore et al., 2011). In a recent study we have shown that inhibition of CXCL4-CXCR3 interaction by an antagonist AMG487 significantly reduced the replication of DENV and JEV, since both viruses use CXCL4 for their rapid replication in host immune cells *in vitro*. The AMG487 treatment reduced the JEV infection in mice and increased the mice survivability suggesting the CXCL4-CXCR3 axis as the therapeutic target for prophylaxis of dengue (Ojha et al., 2019).

IFNs play major antiviral roles. IFN-α/β have proved to be of considerable value in some chronic virus infections, particularly hepatitis and papillomavirus infections (Finter, 1994). A study suggests the anti-viral activity of IFN-α and ribavirin as a combination therapy against DENV (Piresde Mello et al., 2018). Studies also suggest that manipulating IL-10, an anti-inflammatory cytokine may serve as an effective antiviral treatment in addition to the development of a safe dengue vaccine (Tsai et al., 2013).

A live attenuated vaccine known as Dengvaxia, developed by Sanofi, was approved by the Food and Drug Administration (FDA) in the United States licensed in May 2019, for use in children 9–16 years old living in an area where dengue is common such as US territories Samoa, Puerto Rico and Virgin Islands. Another live attenuated vaccine, named TetraVax-DV, developed by Butantan, is currently in clinical trial phase 3 in Brazil (Durbin et al., 2011). The Dengue tetravalent vaccine, developed by Panacea Biotec Ltd, is in phase 2 trial in India (TDB Panacea Agreement, 2017).

## Author Contributions

PG has conceptualized the approach. All authors wrote the article.

## Conflict of Interest

The authors declare that the research was conducted in the absence of any commercial or financial relationships that could be construed as a potential conflict of interest.
